# Changes in Spousal Intimacy in Women Suffering Trauma Symptoms from Domestic Abuse: A Culturally Embedded Intervention Study in Pakistan

**DOI:** 10.3390/ijerph21081045

**Published:** 2024-08-08

**Authors:** David L. Rowland, Mehwish Kamran Ehsan, Stewart E. Cooper

**Affiliations:** 1 Department of Psychology, Valparaiso University, Valparaiso, IN 46383, USA; stewart.cooper@valpo.edu; 2 Department of Professional Psychology, Bahria University, Islamabad 44000, Pakistan; neha-1@live.com

**Keywords:** domestic violence, intimate partner violence, trauma, PTSD, intimacy, physical abuse, sexual abuse, psychological abuse

## Abstract

While emerging research is highlighting the significant effects of culture on marital and family relationships, studies investigating relationship intimacy and abuse in non-Western cultures are non-existent. This investigation assessed relationship intimacy in Pakistani women experiencing trauma symptoms (PTSD) from domestic abuse (DA) who received a culturally informed trauma intervention in a context that differs greatly in values and assumptions about marital relationships relative to Western traditions. Forty women meeting inclusion criteria were assessed on domestic violence type and characteristics (both victim and perpetrator characteristics), PTSD symptomology, and three aspects of relationship intimacy: engagement, communication, and shared friendships. PTSD symptomology and relationship intimacy were reassessed post-intervention. Results indicated significant changes in engagement and communication intimacy following the intervention, with engagement decreasing and communication increasing. The third aspect of intimacy, namely, shared friendships, showed no change. Engagement and overall intimacy showed significant negative correlations with physical abuse, though not with sexual or psychological/emotional abuse. These findings are interpreted within a cultural context where women have few options for leaving an abusive relationship. As such, the results highlight the importance of culture when studying facets of intimate relationships and the need to use culturally informed assessments to better understand the experience of intimacy within abusive relationships.

## 1. Introduction

Although an age-old problem, domestic abuse (DA: now often termed “intimate partner violence” or IPV) has been increasingly recognized as one of the most serious challenges facing women today [1,2]. Specifically, the World Health Organization [WHO] [3] and others [4] have confirmed the widespread and worldwide prevalence of DA, reportedly as high as 60–75% depending on the type of abuse (e.g., spousal, family, sexual) and the region of investigation. Although considered a violation of human rights, the WHO [5] estimates that over one billion women lack legal protection against abuse by a partner or family member.

In Pakistan, prevalence data from probability samples are scant, but studies suggest a fairly high rate of DA. The popular press intimates that Pakistan falls on the higher side of comparison countries [6,7]. In addition, data-based studies using community samples report wide ranges in DA prevalence in Pakistan, for example, 21–50% of women for any type of DA [1], and 50–70% for DA from either the intimate partner or another person over their lifetime [8,9,10]. Even these rates may underestimate the true prevalence of DA, given its covert nature and probable underreporting. DA in Pakistan cannot be attributed to any single factor but is likely linked to any number of factors, including stressful financial conditions, perception of the wife’s infertility, cultural practices, illiteracy, abuser characteristics, and conflict with husband, family, and in-laws [11,12]. Furthermore, a strong patriarchal system that designates lower status to women means that legal protections for female DA victims in Pakistan are often unenforced, weak, or non-existent [8,13]. Pakistan’s problem, however, is not unique to this particular country but reflects a larger problem typical of the sociocultural milieu of central/south Asia [14,15,16].

### 1.1. Posttraumatic Stress Disorder (PTSD) as a Consequence of DA

The effects of DA often first appear as acute stress disorder [17,18] with potential for subsequent development as posttraumatic stress disorder (PTSD) [19,20]. Generally speaking, PTSD arises from a complex interplay of physiological and psychological factors and involves brain regions such as the amygdala, hippocampus, and prefrontal cortex [21], i.e., neural structures mediating emotional processing, memory encoding and consolidation, and fear conditioning and extinction. PTSD can disrupt normal memory processing, leading to fragmented and intrusive memories [22] associated with maladaptive fear conditioning and impaired extinction learning [23]. Pre-existing psychological factors, such as prior trauma exposure, childhood adversity, and personality traits, typically increase vulnerability to developing PTSD, while cognitive processes such as negative appraisals of the trauma and persistent rumination contribute to its maintenance [24]. Finally, genetic factors regulating stress response may also influence an individual’s risk of developing PTSD following trauma exposure [25].

PTSD typically entails the perception of physical or severe psychological threat or injury, including death, as an individual faces a traumatic event [26]. The long-term consequences of such experiences may include traumatic stress, intrusion or re-experiencing, negative alterations in mood or cognitions, and increased arousal symptoms. Acute reactions may manifest in the form of disturbing memories, avoidance behaviors, physical symptoms, general loss of interest, and sleep difficulties [27]. Consistent with these effects, women who experience DA often suffer from PTSD symptoms, exhibiting distrust, guilt, and a sense of betrayal, and showing signs of hypervigilance, avoidance of traumatic triggers, anxiety, re-experiencing through flashbacks, nightmares, and insomnia [28]. Yoshihama and Horrocks [29], for example, demonstrated that women who faced ongoing emotional DA report higher levels of re-experiencing and avoidance symptoms typical of PTSD than those of non-victims.

### 1.2. Spousal Abuse and Feelings of Intimacy

Intimacy—the feeling of being emotionally close and supported—is often considered a bedrock of a stable marital relationship [30,31,32]. Given the often-intimate nature of victim–abuser interactions (hence, “intimate” partner violence), the idea that spousal intimacy might show substantial variation across women experiencing DA may, at first glance, seem unexpected. However, the relationship between victim and abuser is seldom straightforward and is often characterized by a series of both negative and positive interactions. A common but not universal pattern of DA between intimate partners is that of alternation between violent abusive behavior and apologetic heartfelt remorse accompanied with promises of change by the perpetrator [33], a cycle of victim–abuser interactions that has now been well characterized [34].

Episodes of abuse typically constitute a fairly small fraction of the interactions between victim and abuser, with the remainder of the time often characterized by pleasant, intimate, and even superficially normal interactions, particularly strongly reinforced by the abuser when the victim’s behavior conforms with the abuser’s demands for control [35]. As a result, victims of abuse often experience both external and internal reasons for both staying in and leaving a relationship [36]. Specifically, while women who stay in an abusive relationship might do so for external reasons such as financial constraints and impact on children (including fear of loss of custody), internal factors also play a critical role, including ones related to interpersonal dimensions with the spouse/abuser [36,37]. For example, co-dependency, spousal-missing, and fear of loneliness and loss of attachment may discourage women from leaving their relationship, while feelings of emotional closeness, mutual affection, sexual passion, and shared interests and goals may strongly encourage her to stay in the relationship [38,39]. In fact, ongoing, residual, or (even the hope of) a new or restored intimacy may constitute reasons why some women are reluctant to leave an abusive relationship. In this regard, internal factors play out with complexity and often uniquely for each individual and relationship, with women who experience reason to stay in their abusive relationship often displaying distorted thinking that minimizes or denies the problem, that shifts blame for the problem to themselves, or that sympathizes with the abuser [40].

Yet, the existing research literature examining the complex relationship between facets of spousal intimacy and abuse is both scant and, as far as we can discern, largely Western-based, a context that is not particularly representative of women’s experiences in most of the world, where limited options exist for leaving a marriage. While research exploring intimacy and spousal abuse separately in non-Western samples is not uncommon [15,16,41,42,43], studies attempting to link the two are non-existent. In fact, even in Western-based samples, studies linking intimacy and spousal abuse are limited. The findings from one such study [44] generally iterated an expected outcome for intimacy related to abuse: comparing matched samples of abused and non-abused women on sexual intimacy and compatibility, abuse had negative effects on sexual assertiveness, satisfaction, and overall sexual intimacy. Interestingly, the frequency of sexual intercourse was significantly higher in the abused women, a possible compliant strategy designed to avoid abusive episodes. Two other studies address intimacy within abusive relationships, and both highlight the complex and sometimes confusing effects of abuse on intimacy in victim–abuser relationships. For example, one study reported that positive emotions and behaviors by the victim may inhibit dyadic violence in some couples, but self-disclosure appears to have no effect [45]. The second study indicated that while verbal aggression among couples can negatively impact the level of intimacy, quite unexpectedly, levels of intimacy were unrelated to levels of physical assault, sexual coercion, or physical injury, with no explanation for this apparent paradox offered in the available text [46]. In general, however, the scarcity of studies on intimacy in abusive relationships, as well as the varying results in the existing studies, suggests the need for further study regarding a potential connection between DA and intimacy, particularly given that ongoing intimacy—or even the hope for rekindling intimacy—may represent a critical internal justification for women who choose to remain in an abusive relationship. From a non-Western perspective, a further non-negotiable factor for women staying in an abusive relationship may stem from the lack of socially, culturally, and legally viable options for leaving a marriage.

### 1.3. Interventions for DA-Related PTSD

Various approaches have been implemented to address DA against women (including DA in Pakistan), including interventions at the community, family, and individual levels [47]. Community and family interventions, which strive to change attitudes and behaviors, may target community stakeholders, extended families, and spouses/men in order to reduce the overall prevalence of DA [48,49]. Such approaches are critical to developing enduring strategies by increasing the valuation of women by altering deeply embedded sociocultural traditions and practices. Such programs may also lead to further legislative protections for women and eventual reform. Nevertheless, when legalities clash with culture and religion, abuse event reporting and enforcement are often weak or ineffective [50,51]. Furthermore, community and family interventions generally represent long-term strategies aimed at changing attitudes and practices over generations, not months or years. In the meantime, women continue to suffer with PTSD symptoms from DA, and even when/if community programs begin to show results, DA will persist in situations/areas where traditional norms are deeply ingrained. Thus, more immediate individual-level interventions may be warranted to help women manage symptomatology that affects their psychological health and severely disrupts their daily functioning [52].

In general, treatments for PTSD have been both pharmacological and psychological. For example, antidepressants may be effective in managing PTSD, although this approach primarily targets somatic symptoms [53]. Several psychologically based trauma interventions have shown promise [54]. For example, trauma-focused cognitive behavioral therapy (CBT) and eye movement desensitization and reprocessing have garnered encouraging support for PTSD symptom reduction [55], although such psychotherapies often necessitate numerous adjustments in order to make them culturally acceptable and workable in non-Western contexts [56,57]. Given the imagery-based nature of PTSD flashbacks, the use of mental imagery as a complement to verbal-based therapeutic strategies also appears to have had some success in situations where strong language/narrative skills are lacking—such as in cross-cultural contexts—and situations requiring non-native verbal skills or involving limited literacy levels [58,59,60,61,62].

### 1.4. Rationale and Aims

In 2021, Kamran Ehsan and Rowland [58] published an initial study demonstrating that female victims of DA in Pakistan participating in an imagery-based treatment program showed significant reductions in PTSD symptoms. This reduction was notable because, unlike many situations involving spousal abuse, these women were generally not in a position to leave their relationship, and they hence might have experienced continued exposure to abuse. In an attempt to understand changes in relationship dynamics that might provide further context for the reduction in PTSD symptomology, we also assessed changes in marital/dyadic intimacy in these women before and after treatment, thereby providing a parallel and companionate study to the initial investigation.

As noted previously, few studies have directly examined the connection between DA and intimacy, and those that have suggest a varied relationship between them. To the extent that ongoing and/or residual feelings of intimacy may constitute an important factor in the stay/leave decision-making process, we believe the relationship between DA and intimacy deserves deeper exploration. Furthermore, to our knowledge, no studies have investigated *changes* in intimacy associated with a treatment intervention designed to reduce PTSD symptomology related to DA. Finally, studies exploring intimacy and DA have been carried out only within a Western cultural context where women, for the most part, have access to support systems as well as the option to leave the relationship. For such women, the presumption of diminished relationship intimacy is perhaps not particularly surprising [44]. However, such conditions differ radically from women experiencing DA in much of the rest of the world, where social and community support may be non-existent for victims of DA, and where cultural traditions actively discourage and/or prevent women from disclosing abuse and/or attempting to leave the marriage. That is, the “leave” option may be very limited in the stay/leave dilemma for many of these women. Such women, acutely aware that there may be “no way out”, may need to identify other strategies for coping with their situation. For example, they may focus on managing their anxiety and negative emotions and attempt to identify ways to survive within their prescribed social/family roles of spouse, wife, and mother—a situation that may require them to actively manage their feelings toward their spouse as well as to “manage” the relationship itself.

In order to further understand the relationship between intimacy and DA, we conducted an exploratory analysis to assess intervention-based changes in relationship intimacy in women experiencing trauma from DA in a cultural context that differs greatly in values and assumptions about marital relationships relative to Western traditions. Specifically, in the absence of cross-culturally driven theory on intimacy in non-Western populations, we identified three broad aims:

1. To determine marital- and abuse-related predictors of overall relationship intimacy, as well as specific types of intimacy (e.g., communication, engagement) in women experiencing DA (Aim 1);
2. To assess whether a therapeutic intervention for women experiencing trauma symptoms from DA affected overall relationship intimacy as well as specific types of intimacy (Aim 2);
3. To identify predictors that might account for changes in relationship intimacy following a therapeutic intervention (Aim 3).

## 2. Materials and Methods

### 2.1. Participants

Sixty women identified through the outpatient clinics of hospitals (medical/psychiatric/psychological units) located in two major cities in Pakistan, Rawalpindi and Islamabad, were invited through purposive sampling to participate in a study investigating the remediation effects of therapy on PTSD symptoms related to DA. Purposive sampling is a non-random form of sampling where researchers seek out people who possess specific characteristics for their study. Criteria for participation included outpatient status; married and living with their husband; between 18 and 64 years, based on ages of greater DA vulnerability [63]; meeting PTSD criteria by scoring ≥ 50 on the PCL-C; not taking medication or currently under psychotherapy treatment for the PTSD; free of major psychosomatic and psychiatric disorders based on assessment using the General Health Questionnaire (GHQ); free of incapacitating chronic physical illness, mental disabilities, or other anomalies that might interfere with therapy (as determined by medical records, consultation with primary healthcare providers, and a semi-structured interview); and referral/approval from the patient’s attending psychiatrist or psychologist. Of the 60 candidates, 5 women not scoring sufficiently high on PTSD symptomology did not qualify for participation, and 15 women having severe psychiatric or psychosomatic symptomology were referred for more intensive intervention than was offered through this study. The final sample included 40 women (mean age = 34.3; SE = 1.18). To ensure demographic diversity in education level and socioeconomic status, participants were recruited from both public/government-funded and private hospitals in Rawalpindi and Islamabad.

### 2.2. Assessment Instruments

#### 2.2.1. General Health Questionnaire—28

This widely used instrument (Urdu version) screened out participants having major psychiatric and/or psychosomatic disorders, or levels of PTSD symptoms that might require pharmacological or psychiatric intervention, as determined by high standardized assessment scores as well as clinical judgment (https://eprovide.mapi-trust.org/instruments/general-health-questionnaire, accessed on 7 May 2024). Internal reliability for the four subscales in the current study ranged from 0.72 to 0.86.

#### 2.2.2. Demographic Information Form

This form collected information about the participant’s age, education, occupation, years of marriage, number of children, husband’s job and education, family income, family structure, and DA types.

#### 2.2.3. Standard Intake History Form

Using a semi-structured interview format, information was collected about the presenting complaints, prior treatments, family environment, and medical, work, and school histories of the participants.

#### 2.2.4. Karachi Domestic Violence Screening Scale (KDVSS)

The KDVSS, validated Urdu version [64], was used to verify victimization of DA. The instrument consists of 35 items organized into five subscales—physical abuse; psychological abuse; sexual abuse; abuser characteristics; and victim characteristics—with higher scores representing higher levels on the designated subscale. Participants responded to a 4-point scale ranging from 0 to 3 (0 = never; 1 = some; 2 = often; 3 = most of the time). Total scores can range from 0 to 105, with a cut-off of 30 indicating the presence of abuse. The KDVSS has high internal consistency (0.925) and strong test–retest reliability over one month’s time (0.890). Convergent validity (r = 0.899) and discriminant validity (r = −0.927) support the KDVSS as a discriminating tool for measuring the presence and severity of DA committed by husbands against their wives [64].

#### 2.2.5. PTSD Checklist—Civilian Version (PCL-C)

The PCL-C [65] consists of 17 items in which participants indicate how much they have been bothered by a specific symptom over the past month on a 5-point scale (1 = not at all; 5 = extremely) regarding various stressful life experiences. Overall scores range from 17 to 85. Four symptom categories are included—re-experiencing (items 1–5); avoidance (items 6–7); numbing (items 8–12); and hyperarousal (items 13–17)—but only a single overall index is generated. PCL-C use in clinical and non-clinical populations has shown high internal consistency (0.92), test–retest reliability (0.66–0.70), and divergent and convergent validity (0.60–0.80), with optimal cutoff scores of 44 and 50 suggested for diagnosing PTSD among trauma survivors [66,67,68]. The present study used the validated Urdu version [69], which included modifications to focus more on specific events as they related to DA but demonstrated similar internal and test–retest reliability. Consistent with previous research, a cut-off score of 50 was used. Although the language of the PCL does not index symptomology to a specific trauma, its incorporation into the intervention process for DA linked it specifically to events related to domestic abuse.

#### 2.2.6. Personal Assessment of Intimacy in Relationships (PAIR)

The PAIR was used in this study to assess feelings of intimacy prior to and following an imagery-based therapeutic intervention [39]. The PAIR was administered in English with questions asked in real time by the therapist, who ensured comprehension of the question. Participants were given the option of asking for clarification in the local vernacular to ensure clear understanding.

In an initial study, this 36-item instrument yielded individual scores on five intimacy subscales—emotional, social, sexual, intellectual, and recreational—as well as a conventionality scale intended to assess the degree to which an individual attempts to create a good impression (“faking” scale). Reliability analysis yielded a Cronbach’s alpha of 0.70 or higher for each of the scales, and scale scores were positively associated with subscales from similar types of measures, for example, the Locke Wallace Marital Adjustment Scale and the Moos Family Environment Scale. However, subsequent use of the PAIR with clinical samples did not support the above five scales, but rather yielded a three-factor solution broadly characterized as “Engagement”, “Communication”, and “Shared Friendship” [70]. Given the clinical population studied in the current investigation, we opted for the 3-factor subscale groupings as described by Moore et al. [70], with additional detail related to this decision in Section 2.5, Power and Statistical Analysis.

### 2.3. Procedure

This study was approved by the Ethics Committee of Bahria University, Islamabad, Pakistan. Procedural details, benefits, and risks were explained to participants, including the opportunity for PTSD-related therapy at no charge, with participants providing written informed consent prior to this study. As is customary in Pakistani culture, women were permitted to bring along a friend or supportive family member, but all assessment procedures, including interviews, were administered individually to participants. A sole (female) therapist (MKE) carried out the intervention. This therapist was professionally qualified through academic and practical training in a variety of therapeutic procedures (in doctoral training at the time and possessing a master’s degree in clinical psychology), with her services supervised by a trainer certified in the therapy as well as by qualified faculty in her academic training program. The therapist also ensured adherence to the prescribed progression and order of treatment as noted in publications elsewhere [58,71].

An image-based therapy known as eidetic psychotherapy (EPT: [72]) was used for intervention in this study, with the approach and procedure explained in detail elsewhere in an open-access publication [58]. The use of imagery-based therapy, as is performed in EPT, has long been acknowledged as having a role for psychological disorders [60,73], including in the resolution of anxiety, depression, PTSD, phobias, obsessive–compulsive disorder, and eating.

Simply stated, EPT is an insight-based, experiential-oriented therapy that relies on elicitation of mental imagery (“eidetic”) that enables self-examination so the individual can come to understand why she behaves/feels/responds in certain ways [72]. EPT has previously been used with success in the treatment of PTSD as demonstrated through 30 case histories [74]. Imagery-based therapy may offer several advantages over verbal processing in the treatment of PTSD. For example: (1) PTSD itself often evokes strong imagery-based recall of events [75]; (2) imagery is less likely to be influenced by education/literacy than therapies requiring language proficiency, particularly when therapy is carried out in the person’s 2nd or 3rd language; and (3) imagery largely circumvents the cultural biases embedded in linguistic expression [72,76] and therefore may represent a more culturally friendly approach for treatment in non-Western populations. Specifically, in EPT, as the image of the event is formed, the client—under the guidance of the therapist—interacts with it, allowing it to unfold and experiencing its emotional meaning. Such interactions can lead to self-discovery aimed at resolving long-term negative emotional patterns, increasing patients’ self-reliance and insight, and enabling them to recognize somatic and emotional reactions as well as gaps in consciousness [77].

### 2.4. Study Design

This study used a pre–post within-subjects design to determine whether alleviation of DA-related PTSD symptoms and changes in levels of intimacy (PAIR) were associated with the intervention. This study was thus conducted in three phases: pre-intervention, intervention, and post-intervention; to determine the magnitude of change, the last assessment period was carried out 3 months after therapy ended. A detailed presentation of the therapeutic process and content is described elsewhere in open-access format [58].

#### 2.4.1. Pre-Intervention

During this phase, women provided demographic information, underwent a semi-structured intake interview, were screened for psychiatric disorders using the GHQ, and provided information regarding their experience of DA, level of PTSD symptoms, and feelings of intimacy using the KDVSS, PCL-C, and PAIR, respectively. Eligible participants, that is, those not screened out by the GHQ and showing sufficiently high PTSD levels on the PCL-C, were then approved for further participation.

#### 2.4.2. Intervention

The intervention phase commenced 1–3 weeks after pre-intervention and consisted of 40 min sessions spread over 10–12 weeks, with one session usually scheduled each week (8–10 sessions total). At the beginning of each session, participants were verbally (and briefly) assessed for PTSD symptoms (consistent with the PCL statements). Doing so was aligned with best practices based on measurement-based care [78]. When PTSD symptoms fell in the 0–1 range on most items for a given participant, treatment was terminated, accounting for the variable number of treatment sessions across participants. As such, all participants completed the treatment. However, only pre- and post-treatment PCL scores as described below were used in the statistical analysis for this study.

#### 2.4.3. Post-Intervention

Three months after the intervention ended, participants again formally completed the PCL-C and PAIR to assess PTSD symptoms and intimacy. Evaluation was conducted at this particular time for two reasons: (1) given the “30-day time period” as the reference for rating PCL items, sustained change was needed to register as a clinically significant outcome; and (2) to ensure that effects were not fleeting or transient. The therapist also verified that no intervening treatments were utilized during this time.

Data collection was thus embedded in the evaluation process related to treatment, with the therapist (also disclosed to the clients as part of the research team through the informed consent process) managing both the treatment and the data collection before and after treatment.

### 2.5. Power and Statistical Analyses

Sample size for this pre–post design study was based upon four factors: power analysis, availability of participants, availability of resources, and a presumed medium effect size. To meet the following conditions of a two-tailed *p* ≤ 0.05, β = 0.20, d (effect size) = 0.50, the necessary sample size for ANOVA was 32–34. We managed to initially recruit 60 women into this study, with 40 of these women meeting the pre-qualifications for participation. Thus, this study was adequately powered, with the actual effect sizes (eta squared) ranging from medium to large (as presented in the Results Section).

Analyses were carried out with SPSS (IBM Corp. Released 2017. IBM SPSS Statistics for Windows, Version 25.0 Armonk, NY). In an initial exploration of the data, we determined internal reliability scores for the 5- and 3-factor models of the PAIR assessment instrument described previously within our sample of 40 women. In addition, we conducted our own dimension reduction analyses (e.g., PCA or EFA), neither of which aligned with either the 3- or 5-structure factor models identified in earlier research [39,70]. In determining internal reliabilities for the 3- or 5-component solutions, the 3-factor model (engagement; communication; shared friendships; plus the overall composite score) yielded the more promising Cronbach alphas, with three of the four values for the pre-intervention measures 0.35 or higher. Given that Cronbach alphas for the 5-factor model were considerably lower, we ultimately selected the 3-factor model presented by Moore et al. [70] for use in the current study.

Simultaneous-entry linear regression was used to predict PAIR scores prior to therapy, using a combination of variables that included DA attributes (KDVSS scales), trauma symptomology (PCL-C scores), and relevant demographic variables (Aim 1). Changes in relationship intimacy (both overall and for the three subscales symptoms as measured by the PAIR) following intervention were analyzed using repeated measures ANCOVA, with years of marriage included as a covariate (Aim 2). Predictors of changes in relationship intimacy (PAIR scores) following intervention were assessed through regression analysis that drew from theoretically relevant and empirically supported covariates, including “years of marriage” and “level of DA”, as shown in previous research. PCL-C and PAIR change scores (including PAIR subscales) were calculated as the pretest score minus the posttest score. For regression models, VIF scores for all included covariates were under 2.0, indicating low collinearity. Only significant regression models are reported in detail in the Results Section, with notation that other specific models did not reach significance.

## 3. Results

### 3.1. Description of the Sample and Correlations among Study Variables

Table 1 provides basic information about the sample, showing characteristics of the participants, including their diversity in age, education, years of marriage, and socioeconomic class. Table 2 shows pre- and post-intervention PCL scores as well as KDVSS subscale scores. Table 3 shows correlations between predictor covariates and the regression outcome variables (including change in PCL symptoms and change in PAIR subscale scores) following the intervention.

### 3.2. Predictors of Pre-Intervention Intimacy Scores: Overall and Subscales (Aim 1)

For the overall PAIR scores, both physical abuse and psychological abuse correlated significantly with pre-intervention PAIR scores (r_s_ = −0.442 and −0.332, respectively). However, these two variables were themselves collinear (r_s_ = 0.751), so only the stronger of the two (physical abuse) was included in the regression analysis. In our model that included physical abuse, pre-intervention PCL score, and years of marriage, the overall model was significant (R^2^ = 0.196; F(3) = 2.93; *p* = 0.047), but only higher physical abuse was significantly associated with lower overall intimacy β = −0.338; *p* = 0.030) (Supplementary Table S1).

*Regarding PAIR subscales*, for regression on the pre-intervention PAIR engagement subscale, the overall model was significant (R^2^ = 0.234; F(3) = 3.67; *p* = 0.021) and physical abuse was again significantly related to pre-intervention scores, with higher physical abuse associated with lower partner engagement (β = −0.386; *p* = 0.013) (Supplementary Table S2). No covariates were significantly related to pre-intervention communication and shared friendship subscale scores.

**Table 3:** Pearson correlations among KDVSS subscales, demographic variables, and PCL and PAIR change scores.

Variable	Physical Abuse	Sexual Abuse	Years Married	PAIR Change	PCL Change	Engage Change	Comm Change	Friend Change	Age	No. Child
	r (p)	r (p)	r (p)	r (p)	r (p)	r (p)	r (p)	r (p)	r (p)	r (p)
Physical abuse		0.373 (0.029) *	−0.037 (0.949)	−0.034 (0.723)	−0.064 (0.389)	0.008 (0.395)	0.224 (0.151)	0.100 (0.362)	0.066 (0.462)	−0.109 (0.796)
Sexual abuse			−0.098 (0.694)	−0.001 (0.839)	0.231 (0.133)	−0.184 (0.389)	0.121 (0.666)	0.012 (0.836)	0.105 (0.347)	−0.097 (0.958)
Years married				0.031 (0.984)	−0.037 (0.898)	0.037 (0.822)	−0.018 (0.681)	0.161 (0.169)	0.485 (0.001) **	0.540 (0.001) **
PAIR change					−0.067 (0.941)	−0.623 (0.001) **	0.425 (0.001) **	0.313 (0.054) *	0.028 (0.860)	0.194 (0.253)
PCL change						−0.114 (0.329)	0.010 (0.865)	0.029 (0.587)	0.041 (0.414)	0.183 (0.213)
Engage change							−0.209 (0.039) *	−0.057 (0.575)	0.139 (0.701)	0.081 (0.611)
Comm change								0.066 (0.601)	0.006 (0.622)	0.132 (0.698)
Friend change									0.172 (0.197)	0.363 (0.008) **
Age										0.278 (0.103)

* *p* < 0.05; ** *p* < 0.01.

### 3.3. Changes in Intimacy from Pre- to Post-Intervention: Subscale Scores (Aim 2)

ANCOVA was used to test whether overall and subscale (engagement, communication, and shared friendships) pre-intervention PAIR scores differed from post-intervention scores (Table 4). For the overall score and engagement subscale, scores decreased from pre- to post-intervention, indicating a decrease in engagement intimacy (*p* < 0.001, respectively). For the communication and shared friendship subscales, scores *increased* from pre- to post-intervention, though this change was significant only for the communication subscale (*p* = 0.024) and not for the shared friendship subscale (*p* = 0.078).

### 3.4. Predictors of Changes in Intimacy following Intervention (Aim 3)

To determine predictors of changes in the PAIR subscale scores, regression models for each of the subscales included physical abuse and changes in PTSD symptomology (PCL change score) as covariates. The engagement subscale was significant (R^2^ = 0.260; F(2) = 6.49; *p* = 0.004), with higher levels of physical abuse significantly associated with greater decreases in the engagement subscale following the intervention (β = 0.475; *p* = 0.004) (Supplementary Table S3). However, neither covariate predicted changes in the communication and friendship subscales.

**Table 4:** Changes in intimacy subscale scores following an intervention for domestic abuse.

Subscale	Pre	Post		
	M (SD)	M (SD)	*p*	Eta Squared
Engagement	61.7 (3.9)	39.4 (4.3)	<0.001	0.179
Communication	20.6 (2.17)	26.3 (1.69)	0.024	0.127
Shared friendships	7.1 (1.50)	9.0 (1.40)	0.078	0.078

## 4. Discussion

To our knowledge, this study is one of the few assessing changes in relationship intimacy in women with domestic violence-related PTSD symptoms following an intervention protocol. Furthermore, this study is unique in its focus on a non-Western sample where the typical stay/leave options available to most Western women in abusive relationships do not represent viable choices. Our findings not only documented changes in various facets of intimacy following the intervention, but also identified severity of physical abuse as a salient factor in predicting specific changes in engagement intimacy following the intervention. In addition, they highlighted the independence of various aspects of intimacy within the marital/dyadic relationship.

### 4.1. Changes in Intimacy Following Intervention

In a prior analysis [58], we demonstrated that an imagery-based therapy [79,80]) was effective in mitigating PTSD symptoms in women experiencing DA in Pakistan. The current analysis demonstrated that women’s feelings of intimacy toward their husband also changed following this DA-focused intervention. Equally important, changes in intimacy were neither uniform in direction nor size across subscales. Specifically, based on effect sizes (Table 4), women’s *engagement* with their abusive husband decreased greatly following the intervention, while their *communication* increased moderately. Shared friendships with the spouse did not change following the intervention.

Both cultural and contextual factors in this Pakistani sample of women may help explain the changes in intimacy following the intervention. For one, married women victims of DA are, for the most part, an invisible/neglected population in Pakistan, one in dire need of professional assistance yet unable to access it due to women’s financial dependence on their husbands, the cultural stigmatization associated with family members’ use of psychological services, and the lack of clinics prepared to deal with such clients. Thus, this sample represented a unique group of women whose opportunity for, and experience of, counseling intervention was not only out-of-the-ordinary within this cultural milieu, but also undertaken without spousal knowledge or consent. In experiencing this insight-based intervention aimed at elucidating the etiology of the women’s emotional distress, women came to the eventual realization that the behaviors directed toward them by their husbands were indeed “abusive” (as opposed to the common culturally self-negating interpretation of inadequacy by the woman) and that their strong negative emotional response was, in fact, valid under the circumstances. This rare opportunity for catharsis and improved management of emotional responses (without financial burden), likely contributed to the strong positive effects of counseling in alleviating PTSD symptomology. It may also have afforded these women the psychological fortitude not only to better cope with the consequences of the DA [80,81] but also to take steps to actively manage their spousal relationship. It is within the above context that the patterns of change in intimacy, along with role that physical abuse played in those changes, require interpretation.

Specifically, we found that physical abuse emerged as a predictor of intimacy prior to the intervention, with greater physical abuse being associated with lower overall and lower engagement intimacy at the outset of this study. It was also associated with larger decreases in engagement intimacy following the intervention. In this regard, one might argue that as toxic as all the types of abuse are, physical abuse may be the most damaging to an individual’s core life schemas of interpersonal trust, safety, power, and control [82], thereby having a larger effect on certain aspects of intimacy. Accordingly, we [58] and others [83] have found that physical violence and coercion are generally associated with higher PTSD symptoms both at the outset of therapy and following the intervention. Consequently, the lower levels of intimacy associated with physical abuse are both plausible and understandable.

Although physical abuse was more strongly associated with decreased engagement intimacy, the group as a whole displayed a pattern of decreased engagement intimacy following the intervention. Although direct evidence from our study is lacking, we surmise that women may use this decreased engagement (including sexual engagement) as part of a survival tactic that specifically results from their inability to exit the abusive relationship, that is, such a strategy may be strongly intensified in women who, because they must remain in their relationship, need to develop a coping strategy that fits within the restrictions of their situation. In this regard, a strategy of decreased engagement would reflect an active response to an ongoing threat [84]. Although Western-based research has intimated that no spousal (wife) behaviors successfully suppressed partner violence once it had begun [85,86], in strongly patriarchal cultures that prevent or strongly discourage separation or divorce, the decreased engagement strategy may have served as a way of reducing the likely *onset* of partner violence. Indeed, for women who have the option of leaving a relationship, disengagement typically occurs through physical isolation from the abusive spouse and, to a large extent, becomes a moot point. In women having limited or no option to leave, decreased psychological/emotional engagement may have represented both a necessary and self-possessed strategy, given their newly developed confidence as well as skills in stress management and emotional regulation developed during the intervention.

In a manner congruent with increasing personal safety, women showed an increase in communication intimacy following the intervention. Following the logic above, women in cultures where leaving the marriage may be extremely difficult or impossible may consider increased communication as one means of enhancing self-protection. For example, for many situations involving trauma, victims may respond with freezing rather than flight/fight. With the effect of the intervention decreasing the subjective experience of the trauma, a freezing response may have been displaced by an action response, perhaps manifested in this case by increased communication. More (rather than less) information from a potential abuser might enhance preparing for and coping with the challenges and attacks (see also [45]). However, with no covariates emerging as significant predictors of the increased communication following intervention, identification of other possible explanatory variables is needed to provide further understanding of this pattern of change.

### 4.2. Understanding the Lack of Covariation in Intimacy with PTSD Score: A Cultural Integration

One unexpected finding from this study was the lack of an association between changes in PTSD symptomology and changes in intimacy scores. Over the course of the intervention, PTSD symptoms were reduced at the same time that engagement intimacy decreased and communication increased. However, despite the concomitant changes, the changes were not correlated. The lack of an expected collinearity between changes in PTSD symptomology and changes in intimacy may be attributed to several factors, including methodological and cultural issues, some of which may have been intertwined.

In terms of methodological issues, we identify three possible factors: First, the relatively low sample size of 40 participants may have been insufficient for patterns between these two domains to emerge. Second, previous studies have found that the underlying structure of the PAIR instrument that emerged for clinical (distressed) samples revealed a completely different factor structure from the original five factors developed on a non-clinical, non-distressed population. Third, our methodology—which selected women high in PTSD symptomology (and thus limited the range of scores to the upper quadrant of the distribution)—may have resulted in a truncated distribution that obscured the emergence of a true relationship between these variables; that is, had a greater range of PCL (PTSD) scores been available within the data set, an association might have emerged. In addition, women with very high PTSD symptomology may have been screened out by their attending psychiatrist as a “patient-protective” measure.

Regarding cultural issues, items and subscales of the PAIR assessment instrument suggest a significant level of Western bias. Specifically, while the PAIR items may capture important facets of intimacy in Western cultures, marital intimacy in non-Western cultures may manifest very differently, given the different cultural views and expectations toward sex roles, partner selection/marriage, family structure, education, sexuality, and reproduction (e.g., [87,88]). A potentially useful lens is the current value on “companionate” marriage in western societies vs. “traditional” marriages in much of the rest of the world [89]. Thus, PAIR questionnaire items related to recreational and/or intellectual intimacy may have low relevance/value in assessing marital relationships in many non-Western cultures. Given the above, it is not surprising that the intimacy constructs assessed by the PAIR instrument in our sample did not demonstrate particularly high levels of internal consistency. Indeed, the correlations among PAIR pre-intervention subscale scores were not only low but actually negative among some subscales. Such patterns not only indicate the likely orthogonal nature of intimacy constructs [90], but also iterate the importance of exploring research questions in non-Western samples where the assumptions underlying instrument development, characteristics, and use may not share the same level of validity as for Western-based samples [91]. Thus, while the individual subscales showed moderate validity (and utility) within our study, the instrument did not function as a unified measure of intimacy. Such issues suggest the critical need to develop culturally informed measures of intimacy, as culture undoubtedly has a large influence on the particulars of how intimacy manifests within marital contexts.

Finally, as noted previously, a major challenge of this study was the need to circumvent issues of ongoing abuse over the course of treatment, that is, therapist–client discussions related to incidents of (ongoing) abuse were generally avoided, given that unpredictable consequences might have resulted had spouses or family members become privy to such dialogue. Hence, therapy focused strongly on coping with emotional fallout [92] through treatment alliance, skill building, emotional catharsis and regulation, self-care, and education, that is, processes largely consistent with the first stage of treatment for complex trauma as described in the literature [93,94].

Given this particular focus of the treatment, issues regarding spousal intimacy were not specifically targeted as part of the treatment protocol—rather, they occurred as a collateral byproduct of the therapeutic process. Thus, we recognize the need for longer-term follow-up to ascertain whether changes in relational intimacy following the intervention were sustained over a longer time interval and, as importantly, whether this response was actually beneficial or, alternatively, counterproductive to the women’s situations. Such information would help assess whether intimacy issues might be addressed more intentionally within the context of treatment of women for DA who are unable to vacate the abusive relationship.

### 4.3. Strengths and Limitations

Few studies have attempted to track changes in intimacy associated with an intervention for women suffering from DA in a sample characterized by diversity in socioeconomic status, education, age, and origin (rural vs. urban). Our study highlights the multidimensional aspects of intimacy for women undergoing treatment, demonstrating the complex interplay between the need for emotional disengagement while yet maintaining a sense of physical and psychological protection and security from further abuse. It further clarifies the importance of cultural context when studying intimacy in relationships. For example, women participating in this intervention did not have the option to exit their relationship, thereby placing boundaries on off-limit topics and life strategies. Moreover, it illustrates that specific components of Western-conceptualized intimacy may have diminished relevance or meaning in systems that attribute different gender and marital roles within dyadic (reproductive) relationships. In conjunction with this latter point, this study demonstrates the dire need for the development of culturally fitting measures of intimacy and its possible subscales.

We are acutely aware of this study’s limitations. Besides the potential issue regarding the use of a Western-based assessment tool as a valid measure of intimacy in our sample, several other limitations deserve mention: First, as a real-world clinical study designed to aid a population in need under conditions of limited treatment resources, the study methodology relied on a sample of convenience rather than a randomized, controlled trial. As such, the extent to which changes in intimacy could be attributed specifically to the intervention are limited. Furthermore, we presume that any real effects may not have been specific to the type of therapy used in this study, but rather that *other* types of therapy or aspects of the therapeutic process might have been associated with changes in intimacy. Nevertheless, although it was not clear what factors (other than physical abuse) help explain the observed changes in intimacy, the fact that women who, on average, had suffered DA for years showed changes in relationship intimacy over the course of 10–12 weeks of therapy strongly argues that those changes were not merely the result of the passage of time.

Although a limited sample size (and thus limited power) restricted our ability to identify a viable factor structure, we note that a sample size of 40 women is considerable for an intervention study. Indeed, our sample size exceeded that of other studies on women suffering complex trauma from DA who participated in a therapeutic intervention (e.g., [93]) and was sufficiently powered to detect pre–post differences. Furthermore, as sample size is often constrained by factors such as practical feasibility, patient access, and sufficient resources [95,96,97], the current study may be best viewed as a proof-of-concept trial (e.g., [98]), demonstrating that psychological intervention may impact relationship intimacy in women who have suffered DA. In retrospect, this study would have benefitted from the inclusion of an untreated or waitlisted control group and the addition of further measures of intimacy as well as other potentially relevant covariates, such as select co-morbidities, frequency of ongoing abuse, relationship quality, and other environmental social stressors.

## 5. Conclusions

This study highlighted the multidimensional aspects of intimacy for women undergoing treatment for PTSD symptoms resulting from domestic abuse. Intervention resulted in decreased engagement intimacy and increased communication intimacy, demonstrating the complex interplay between the need for emotional disengagement while yet maintaining a sense of physical and psychological protection and security from further abuse. Level of physical abuse was most strongly associated with intimacy changes following the intervention. This study further highlighted the importance of cultural context when studying intimacy in relationships, particularly in complex situations involving domestic abuse.

## Figures and Tables

**Table 1:** Frequencies (*n*) and related percentages (%), or mean and standard deviation for the demographic characteristics of participants (*n* = 40).

Variables	Mean ± SD or Categories	*n*	%
Age	34.3 (7.47)		
Education	Secondary Intermediate Graduate Postgraduate	9 7 11 13	22 17 27 32
Occupation	Housewife Employed	25 15 3 9 10 18	62 37 7 22 25 45
Monthly income	20,000–30,000 (USD 119–179) 31,000–50,000 (USD 185–298) 51,000–60,000 (USD 304–358) 61,000-above (USD 364–above)		
Family system	Nuclear	16	40
	Extended	24	60
No. of children	1.8 (1.22)		
Years of marriage	8.8 (6.49)		
Marital status	First marriage	35	87
	Second marriage	5	12
Residential area	Lower class	5	12
	Middle class	15	37
	Upper middle class	11	27
	Upper class	9	22

**Table 2:** Scores on PCL (pre- and post-intervention) and KDVSS subscales (pre-intervention only).

Variable	Mean	Range	Std. Deviation	Cronbach Alpha
Pre-PCL	60.9	50–78	8.70	0.89
Post-PCL	19.8	17–22	1.39	0.44
Physical abuse	12.7	8–15	2.23	0.62
Sexual abuse	13.4	6–22	2.34	0.70
Psychological abuse	38.8	24–44	4.55	0.73
Abuser traits	13.3	6–22	2.38	0.69
Victim traits	14.0	9–25	2.30	0.48

## Data Availability

Output data are available from MKE upon reasonable request.

## Supplementary Materials

The following supporting information can be downloaded at: https://www.mdpi.com/article/10.3390/ijerph21081045/s1, Table S1. Regression of Pre-PCL scores, years of marriage, and Physical Abuse on the outcome variable, Pre-intervention overall PAIR score; Table S2. Regression of Pre-PCL scores, years of marriage, and Physical Abuse on the outcome variable, Pre-intervention Engagement score; Table S3. Regression of PCL change scores and Physical Abuse on the outcome variable, PAIR Engagement change score.
