# Supplementary material for: Changes in Spousal Intimacy in Women Suffering Trauma Symptoms from Domestic Abuse: A Culturally Embedded Intervention Study in Pakistan
Source: Int J Environ Res Public Health. 2024 Aug 8;21(8):1045. doi: 10.3390/ijerph21081045 (PMC11353746; doi:10.3390/ijerph21081045)
Supplement: Supplementary file 1 [file ijerph-21-01045-s001.zip › ijerph-3078107-supplementary.pdf]

SUPPLEMENTARY TABLES 1-3

Supplementary Table S1. Regression of Pre-PCL scores, years of marriage, and Physical Abuse on the outcome variable, Pre-intervention overall PAIR score

Variable	b	Std error	Beta	t-value	p-value
Constant	93.67	7.76		12.08	<.001
Pre-PCL score	0.061	0.089	0.105	0.690	.495
Years Marriage	0.928	0.684	0.203	1.357	.183
Physical Abuse	-0.823	0.347	-0.321	-2.371	.023

Supplementary Table S2. Regression of Pre-PCL scores, years of marriage, and Physical Abuse on the outcome variable, Pre-intervention Engagement score

Variable	b	Std error	Beta	t-value	p-value
Constant	64.74	5.82		11.14	<.001
Pre-PCL score	0.057	0.067	0.127	0.858	.397
Years Marriage	0.792	0.513	0.225	1.542	.132
Physical Abuse	-0.677	0.260	-0.386	-2.603	.013

Supplementary Table S3. Regression of PCL change scores and Physical Abuse on the outcome variable, PAIR Engagement change score

Variable	b	Std error	Beta	t-value	p-value
Constant	32.81	7.48		4.387	<.001
PCL-change	-0.149	0.107	-0.199	-1.390	.173
Physical Abuse	1.320	0.426	0.442	3.096	.004
